# Comparison of dark-field chest radiography and CT for the assessment of COVID-19 pneumonia

**DOI:** 10.3389/fradi.2024.1487895

**Published:** 2025-01-14

**Authors:** Florian T. Gassert, Henriette Bast, Theresa Urban, Manuela Frank, Felix G. Gassert, Konstantin Willer, Rafael C. Schick, Bernhard Renger, Thomas Koehler, Alexandra Karrer, Andreas P. Sauter, Alexander A. Fingerle, Marcus R. Makowski, Franz Pfeiffer, Daniela Pfeiffer

**Affiliations:** ^1^ Department of Diagnostic and Interventional Radiology, School of Medicine & Klinikum Rechts der Isar, Technical University of Munich, Munich, Germany; ^2^ Chair of Biomedical Physics, Department of Physics, School of Natural Sciences, Technical University of Munich, Garching, Germany; ^3^ Munich Institute of Biomedical Engineering, Technical University of Munich, Garching, Germany; ^4^ Munich Institute for Advanced Study, Technical University of Munich, Garching, Germany; ^5^ Philips Innovative Technologies, Hamburg, Germany

**Keywords:** COVID-19, radiography, dark-field, pneumonia, lung imaging

## Abstract

**Background:**

Dark-field chest radiography allows the assessment of the structural integrity of the alveoli by exploiting the wave properties of x-rays.

**Purpose:**

To compare the qualitative and quantitative features of dark-field chest radiography in patients with COVID-19 pneumonia with conventional CT imaging.

**Materials and methods:**

In this prospective study conducted from May 2020 to December 2020, patients aged at least 18 years who underwent chest CT for clinically suspected COVID-19 infection were screened for participation. Inclusion criteria were a CO-RADS score ≥4, the ability to consent to the procedure and to stand upright without help. Participants were examined with a clinical dark-field chest radiography prototype. For comparison, a healthy control cohort of 40 subjects was evaluated. Using Spearman's correlation coefficient, correlation was tested between dark-field coefficient and CT-based COVID-19 index and visual total CT score as well as between the visual total dark-field score and the visual total CT score.

**Results:**

A total of 98 participants [mean age 58 ± 14 (standard deviation) years; 59 men] were studied. The areas of signal intensity reduction observed in dark-field images showed a strong correlation with infiltrates identified on CT scans. The dark-field coefficient had a negative correlation with both the quantitative CT-based COVID-19 index (*r* = −.34, *p* = .001) and the overall CT score used for visual grading of COVID-19 severity (*r* = −.44, *p* < .001). The total visual dark-field score for the presence of COVID-19 was positively correlated to the total CT score for visual COVID-19 severity grading (*r* = .85, *p* < .001).

**Conclusion:**

COVID-19 pneumonia-induced signal intensity losses in dark-field chest radiographs are consistent with CT-based findings, showing the technique's potential for COVID-19 assessment.

## 1 Introduction

Since the worldwide spread of the severe acute respiratory syndrome coronavirus 2 (SARS-CoV-2) in early 2020, it has held the world in a tight grip and led to a global medical, social, and economic crisis (1). In this context, detection of a COVID-19 infection and assessment of the extent of pulmonary involvement are essential pillars of an effective treatment. While PCR-testing is the gold standard for diagnosis (2, 3), CT imaging is often used in daily clinical routine to assess COVID-19-associated pulmonary pathologies. CT imaging has a high sensitivity for ground glass opacities but comes with a comparably high radiation dose. Conventional radiography, on the other hand, comes with lower radiation exposure. However, it also yields a lower sensitivity for COVID-19-associated lung changes compared to CT imaging (4).

In 2008, grating-based dark-field x-ray imaging was introduced (5). The technique holds promise for the assessment of micro-structural changes in lung parenchyma, as it has been shown to be beneficial for the imaging of various lung diseases in animal models (6–16), including pneumonia (17). Ever since, it has been subject to continuous improvement, optimization, and upscaling from animal models to humans. In 2021, the application of dark-field imaging in human patients was reported for the first time (18–20). In contrast to conventional x-ray imaging, which measures the attenuation of x-rays in the specimen, dark-field x-ray contrast is related to small-angle scattering of x-rays at material interfaces. Dark-field x-ray imaging might provide a new diagnostic tool for the assessment of COVID-19-associated lung changes while exposing the patient to a comparably low amount of radiation (21, 22).

Therefore, the aim of this study was to evaluate the potential of dark-field chest radiography for the detection of COVID-19 pneumonia in humans compared to CT imaging.

## 2 Materials and methods

### 2.1 Participants

Prior to the study, institutional Review Board (IRB) as well as national radiation protection agency approval was obtained (Ethics Commission of the Medical Faculty, Technical University of Munich, Germany; 587/16 S and 116/20 S). All patients gave their written informed consent before enrollment in the study.

#### 2.1.1 COVID-19 patients

Screening took place between May 2020 and December 2020. Adult patients who underwent chest CT at our institution as part of their diagnostic evaluation and were clinically suspected of having a COVID-19 infection were considered for study participation. All CT scans of potential participants were reviewed for COVID-19-related lung alterations by two of three radiologists (FTG, APS, AAF) directly after the imaging, following the CO-RADS assessment criteria for patients suspected of having COVID-19 (23).

This study included only patients who were categorized as CO-RADS 4 (suspected COVID-19), 5 (typical COVID-19 presentation), or 6 (RT-PCR confirmed SARS-CoV-2, if tested prior to the CT scan). Additional criteria for inclusion were the capacity to give informed consent, stand unaided, and hold their breath for 7 s. Patients meeting these criteria were approached immediately following their CT scan.

**Figure 1:**
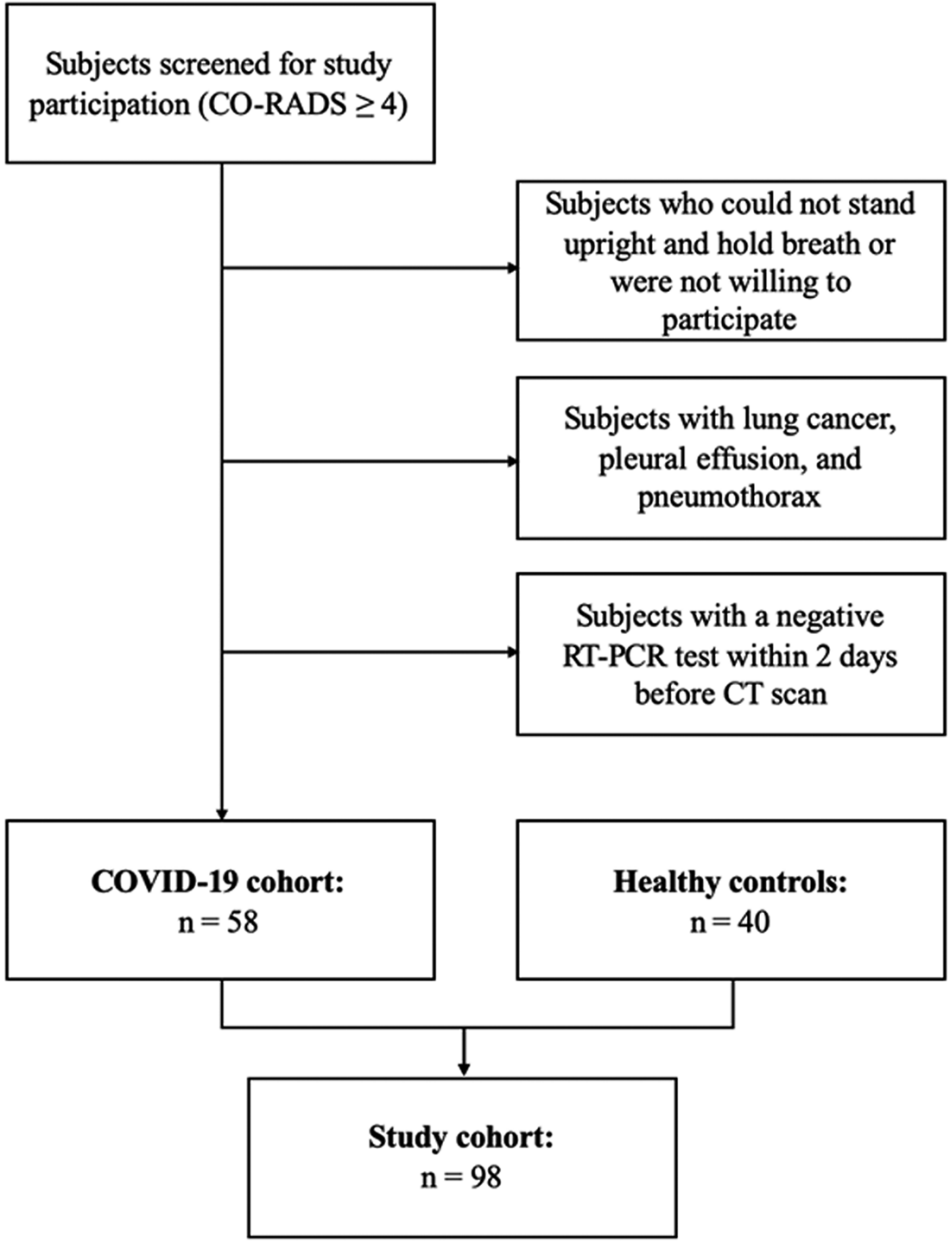
Flowchart illustrating subject selection.

Exclusion criteria included a negative RT-PCR test within 2 days prior to the CT scan, pregnancy, lung cancer, pleural effusion, and pneumothorax. The selection process is detailed in Figure 1.

#### 2.1.2 Controls

From October 2018 to January 2020, adult patients who underwent chest CT scans at our institution as part of their diagnostic assessment were considered for participation in this study. Three radiologists (FTG, APS, AAF) evaluated all CT scans of potential participants for any abnormal lung findings. To be included, patients needed to have a normal chest CT scan, be capable of giving informed consent, and be able to stand upright without assistance. Patients meeting these criteria were contacted immediately after their CT scan. Exclusion criteria included pregnancy, severe health conditions, and any lung abnormalities such as cancer, pleural effusion, atelectasis, emphysema, infiltrates, ground-glass opacities, or pneumothorax. The control group consisted of 40 patients, who have been previously reported in (19).

### 2.2 Dark-field imaging

The dark-field images were acquired with a prototype system for dark-field chest radiography situated at (TUM University Hospital Klinikum Rechts der Isar). This prototype utilizes a standard imaging system equipped with a diagnostic x-ray tube (MRC 200 0508 ROT-GS 1003; Philips Medical Systems) set to 70 kVp and a flat-panel detector (PIXIUM 4343 F4; Trixell) as described previously (18). A three-grating interferometer positioned in the beam path allows for the simultaneous capture of conventional attenuation radiographs and complementary dark-field images (5).

Each participant was imaged posteroanterior at full inspiration while standing upright. The acquisition time of an image was about 7 s, causing an effective dose (21) (participant collective median of all participants) of 41.9 µGy.

### 2.2 CT imaging

CT scans were carried out on one of three different scanners (Philips iCT, Siemens SOMATOM, or Philips IQon Spectral CT) using standard clinical procedures with the following parameters: collimation, 128 mm × 0.6 mm and 64 mm × 0.6 mm; pixel spacing, 0.4 and 0.3 mm; pitch factor, 0.8 and 0.9; peak tube voltage, 120 kVp; modulated tube current, 102–132 mA. The images were then reformatted to a slice thickness of 3 mm, utilizing a convolution kernel specifically designed for lung imaging.

### 2.3 Image evaluation

All readings were performed using a PACS system (Sectra IDS7, Linköping, Sweden) and authorized monitors.

#### 2.3.1 Dark-field radiographs

Four radiologists (FTG, APS, AAF, DP) with different levels of experience in dark-field imaging (3, 6, 8, 10 years) assessed the dark-field radiographs for all participants. All readers were blinded to the group affiliation of images and images were presented in random order. All dark-field radiographs were displayed with the same window level and window width, where black corresponds to a value of 0 and white corresponds to an intensity of a dark-field signal of 0.8 or higher. The readers were asked if COVID-19 pneumonia was present (rated as “1”) or not (rated as “0”) in the following zones: right apical/upper zone, right mid zone, right lower zone, left apical/upper zone, left lower zone. The total dark-field score was the sum of the individual zonal ratings, ranging from 0 (no presence of COVID-19) to 5 (COVID-19 present in every zone).

For quantitative analysis of dark-field images, the dark-field coefficient was calculated for the entire lung according to Gassert et al. (19): In brief, the total dark-field signal of the lung was divided by the lung volume which was derived using the approach from Pierce et al. (24).

For eight COVID-19 patients (three men and five women), the lateral image could not be acquired due to the patient's condition and therefore determination of the lung volume and the dark-field coefficient was not possible. These patients were not included in the quantitative analysis.

#### 2.3.2 CT images

Three radiologists (FTG, FGG, APS) with different levels of experience in thoracic imaging (3, 3, 8 years) assessed the presence of COVID-19 pneumonia in all CT images. The readers were allowed to adjust window and level settings at their convenience. They were asked to rate each of the five lung lobes on a scale from 0 to 5, where 0 meant no involvement, 1 indicated involvement of less than 5%, 2 for 5%–25%, 3 for 26%–49%, 4 for 50%–75%, and 5 for more than 75% (25). The total CT score was the aggregate of the scores for each lobe, ranging from 0 (no involvement) to 25 (complete involvement).

**Figure 2:**
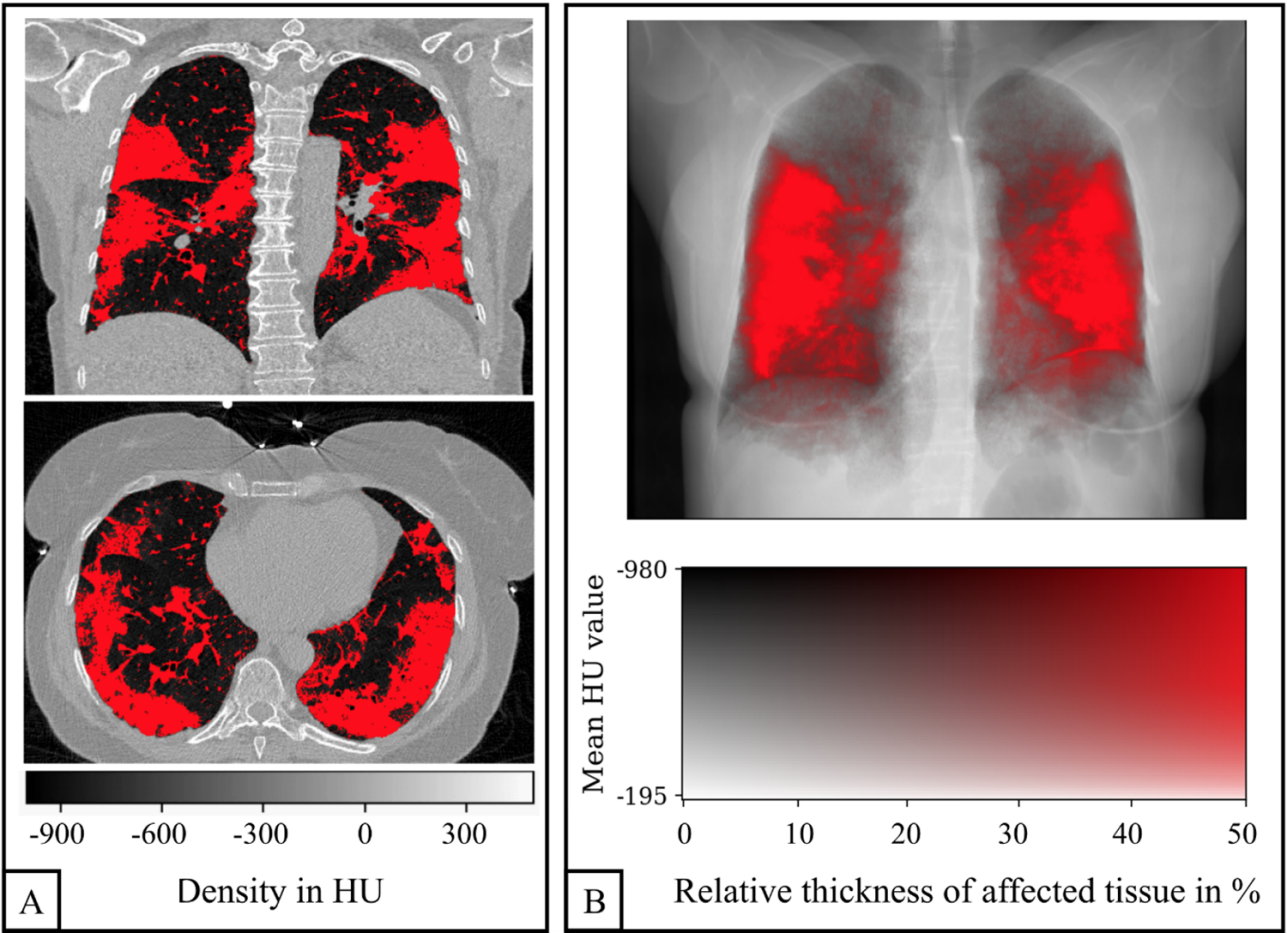
Quantification of COVID-19 pneumonia in a CT scan of a 58-year-old woman. Example slices in axial and coronal reformation are shown in **(A)**. All voxels within the lung with a density greater than −740 HU are labeled as affected (red). The three-dimensional COVID-19 map is projected along the sagittal axis in order to generate an overlay of the CT-based attenuation and the COVID-19 pneumonia projection **(B)**.

Similar to quantitative emphysema assessment, the entire lung was segmented from the chest CT data and a threshold was manually chosen for each COVID-19 patient (range: −810 HU to −600 HU; median: −740 HU) and applied to distinguish between healthy lung tissue and COVID-19-associated changes (Figure 2). The percentage of area with COVID-19-associated changes was defined as the quantitative CT COVID-19-index (COV-I). For calculating the COV-I of healthy participants the threshold was set to −600 HU. Note that the bronchovascular bundle was not excluded from segmentation in both COVID-19 patients and healthy controls and therefore the COV-I of healthy controls was expected to be greater than zero.

To facilitate a visual comparison between the affected regions in CT and planar dark-field images, the three-dimensional CT COVID-19 map was projected along the sagittal axis. This created an overlay of the CT attenuation image and the COVID-19 projection, akin to the emphysema projections described by Urban et al. (20) (Figure 2). A two-dimensional color map was employed, where each pixel's color represented its quantitative COV-I and its brightness indicated attenuation. The intensity of the red color reflects the proportion of voxels affected by COVID-19 relative to the subject's maximum lung thickness, with saturation reached when the ratio of affected tissue thickness to maximum lung thickness hits 50%.

### 2.4 Statistical analysis

Statistical analysis was performed with Python (version 3.6.9), specifically using the packages NumPy (version 1.19.5) and SciPy (version 1.5.0), and R (version 4.2.0), specifically using the package “irr” (version 0.84.1). A *p*-value of < .05 was considered to indicate statistical significance. Using Student's *t*-test, the participant parameters age, weight, and lung volume and the determined COV-I were tested for significant differences between participants with COVID-19 pneumonia and the control group. For the parameter “sex”, a *χ*^2^ test was used. For each participant, the median of the reading-related quantities (total visual dark-field score, CT score, and total visual CT score) from the different readers was used for further analysis. Using Spearman's correlation coefficient, correlation was tested between dark-field coefficient and CT-based COV-I as well as visual total CT score and between visual total dark-field score and visual total CT score. The R package spearmanCI was used to calculate the confidence intervals (CI) for each correlation coefficient (26). The inter-rater agreement was calculated using Fleiss' Kappa. The zone-based ratings for the presence of COVID-19-associated changes were grouped according to the CT-based visual COVID-19 severity gradings of the associated participants. The resulting distributions were tested for significant differences among each other using Fisher's exact test for MxN contingency tables (27).

## 3 Results

### 3.1 Participants

We studied 98 participants (59 men, 39 women), including 58 patients with COVID-19 pneumonia and 40 healthy controls. The average age of the participants in this study was 58 ± 14 [standard deviation] years, and the average weight was 79 ± 16 kg (Table 1). No differences were found between healthy controls and patients with COVID-19 pneumonia regarding sex, age, weight, and total lung volume.

**Table 1:** Subject demographics.

Parameter	All	Healthy	COVID-19	*p*-value
Number of participants	98	40	58	
Men/Women	59/39	25/15	34/24	*p* = .70
Age (years)	58 ± 14	61 ± 12	57 ± 15	*p* = .15
Weight (kg)	79 ± 16	79 ± 15	79 ± 16	*p* = .84
Total lung volume (L)	6.8 ± 1.8	6.8 ± 1.4	6.7 ± 2.1	*p* = .81

Values are given as mean ± standard deviation. *P*-values for the significance testing of differences between the COVID-19 group and the healthy controls are listed in the very right column. Determination of the total lung volume was adapted from Pierce et al. (24) The 40 healthy subjects were also included in Gassert et al. (19).

### 3.2 Dark-field chest radiography qualitative analysis

**Figure 3:**
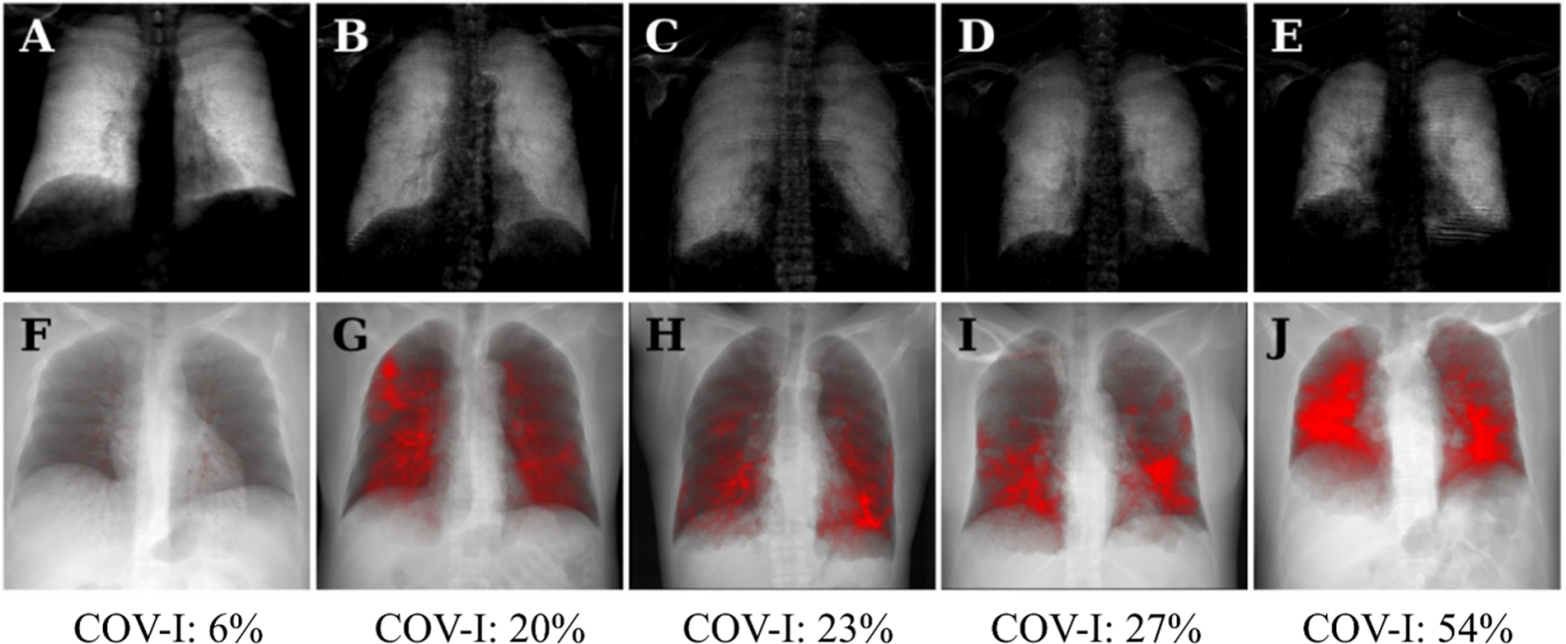
Dark-field radiographs **(A–E)** and projections of CT-based COVID-19 pneumonia quantification **(F–J)** in a healthy, 30-year-old man **(A,F)** and four patients (three men, one woman) with COVID-19 pneumonia **(B–E, G–J)**; respective COVID-19 index below. The same window and level settings were applied within each modality. Conventional radiographs reveal typical opacities in lung regions affected by COVID-19-associated changes, such as predominantly in the right upper field in the first COVID-19 patient **(B)**. While the dark-field chest radiograph of the healthy subject exhibits a strong homogeneous dark-field signal **(A)**, the dark-field signal intensity of the patients with COVID-19 pneumonia appears decreased overall and exhibits a regionally inhomogeneous patchy pattern **(B–E)**, corresponding well to the affected areas in the CT-based projections (red overlay, **G–J**). The red overlay in the CT-based projection of the healthy participant **(F)** corresponds to the bronchovascular bundle.

**Figure 4:**
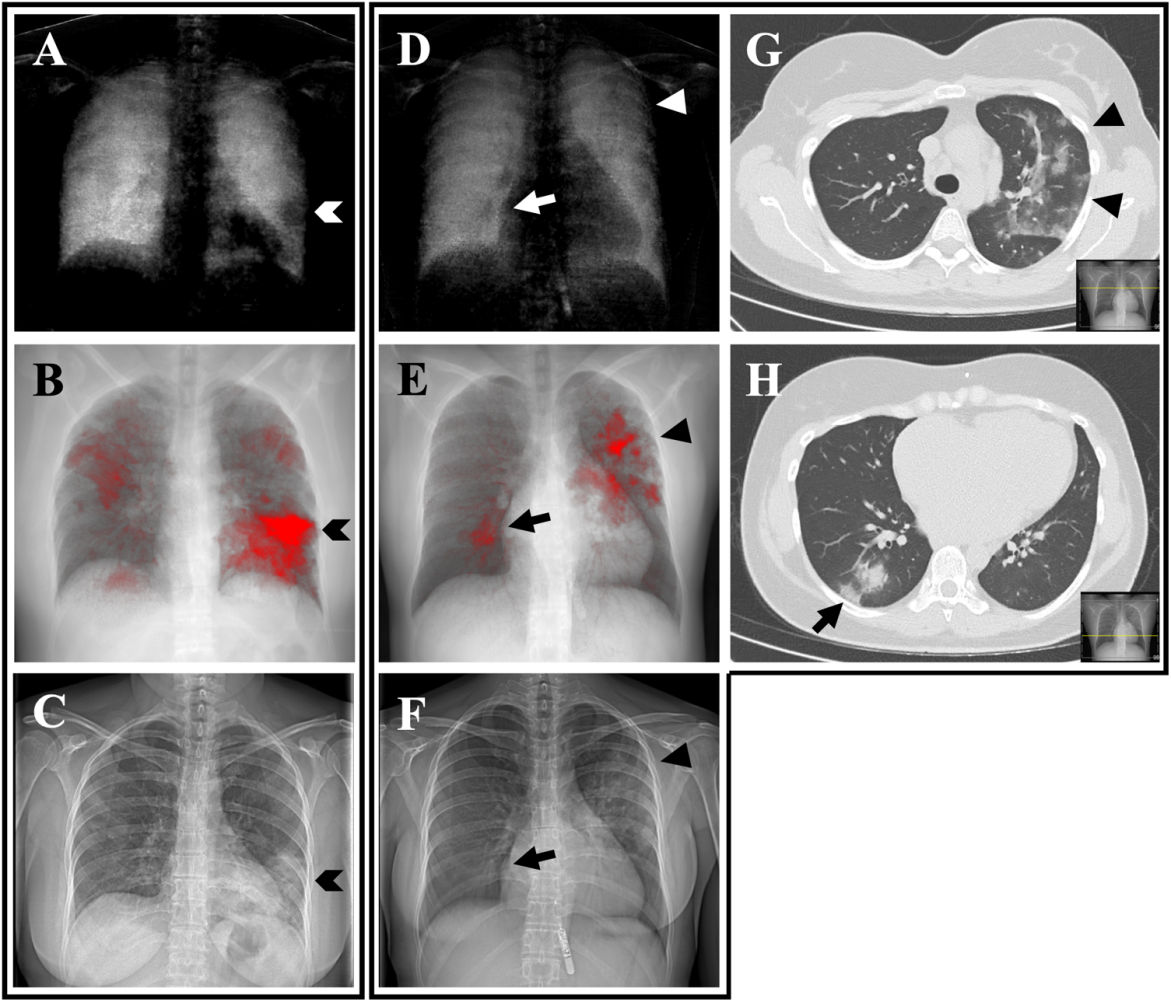
Dark-field radiographs **(A,D)**, CT-based projections **(B,E)**, conventional radiographs **(C,F)** and axial reformations **(G,H)** of a CT scan from a 47-year-old woman **(A–D)** and a 36-year-old woman **(D–F, G,H)**. In the participant on the left, the focal signal loss in the periphery of the left lower zone (**A**, arrowhead) corresponds well to the red area in the CT-based projection (**B**, arrowhead) and the opacity in the conventional radiograph (**C**, arrowhead). The same applies for the second patient **(D–F)** with focal signal loss in the right lower zone (arrow) and the left upper zone (triangle). The corresponding axial CT reformations **(G,H)** reveal opacities in the respective regions.

Figure 3 shows dark-field chest radiographs and CT-based projections of a 30-year-old man without COVID-19 pneumonia (COVID-19-index = 6%) and four example patients with COVID-19-pneumonia. The healthy participant exhibits a strong and homogeneous dark-field signal. The dark-field signal of the patients suffering from COVID-19 pneumonia on the other hand is generally lower and appears to be less homogeneous (Figure 3). While in the CT-based projection of the healthy subject only the bronchovascular bundles are marked in red, red areas in the CT-based projection of the patient with COVID-19 pneumonia also mark COVID-19-associated lung changes. The local signal reduction in participants with COVID-19 pneumonia shows correspondence with CT (Figures 3, 4).

### 3.3 Quantitative analysis

Patients with COVID-19 pneumonia had a higher CT-based COVID-19-index (Cov-I: 25 ± 11%, 95% CI [21.7%, 27.3%]) compared to healthy controls (Cov-I: 5 ± 1%, p < .001, 95% CI [4.7%, 5.5%]). The total visual COVID-19 severity grading from CT was also higher for patients with COVID-19 pneumonia (median: 10; healthy controls: 0; *p* < .001). The inter-rater reliability ranged from .42 to .55 for dark-field readings and from .48 to .60 for CT readings (Supplementary Table 1).

The quantitative CT-based COV-I was positively correlated with the total CT score for visual COVID-19 severity grading (*r* = .91, *p* <.001, 95% CI [.90, .95]) and with the total visual dark-field score (*r* = .76, *p* <.001, 95% CI [.75, .85]).

**Figure 5:**
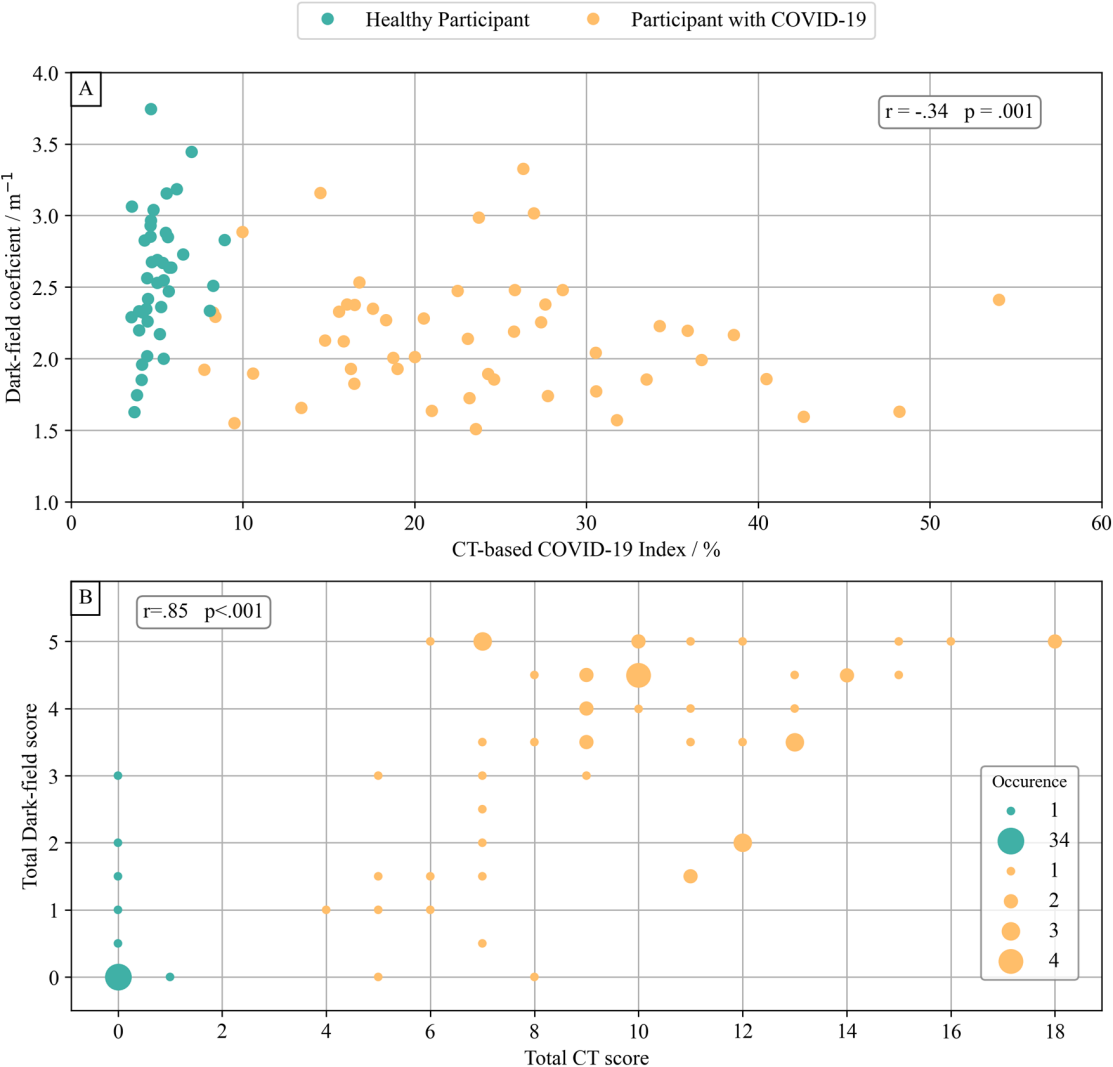
Dark-field-based and CT-based COVID-19 evaluation. The dark-field coefficient was calculated by normalizing the integral of the dark-field signal of each subject's lung area with their lung volume. **(A)** Comparison of dark-field coefficient with the COVID-19 index from quantitative CT evaluation. There was a weak correlation (*r* = −.34, *p* = .001) between dark-field coefficient and quantitative COVID-19 index from quantitative CT evaluation. **(B)** Comparison of total visual rating of the presence of COVID-19 from dark-field images and total visual COVID-19 severity grading from CT. There was a very strong correlation (*r* = .85, *p* < .001) between total visual COVID-19 presence in dark-field radiographs and total CT-based visual COVID-19 severity grading.

**Figure 6:**
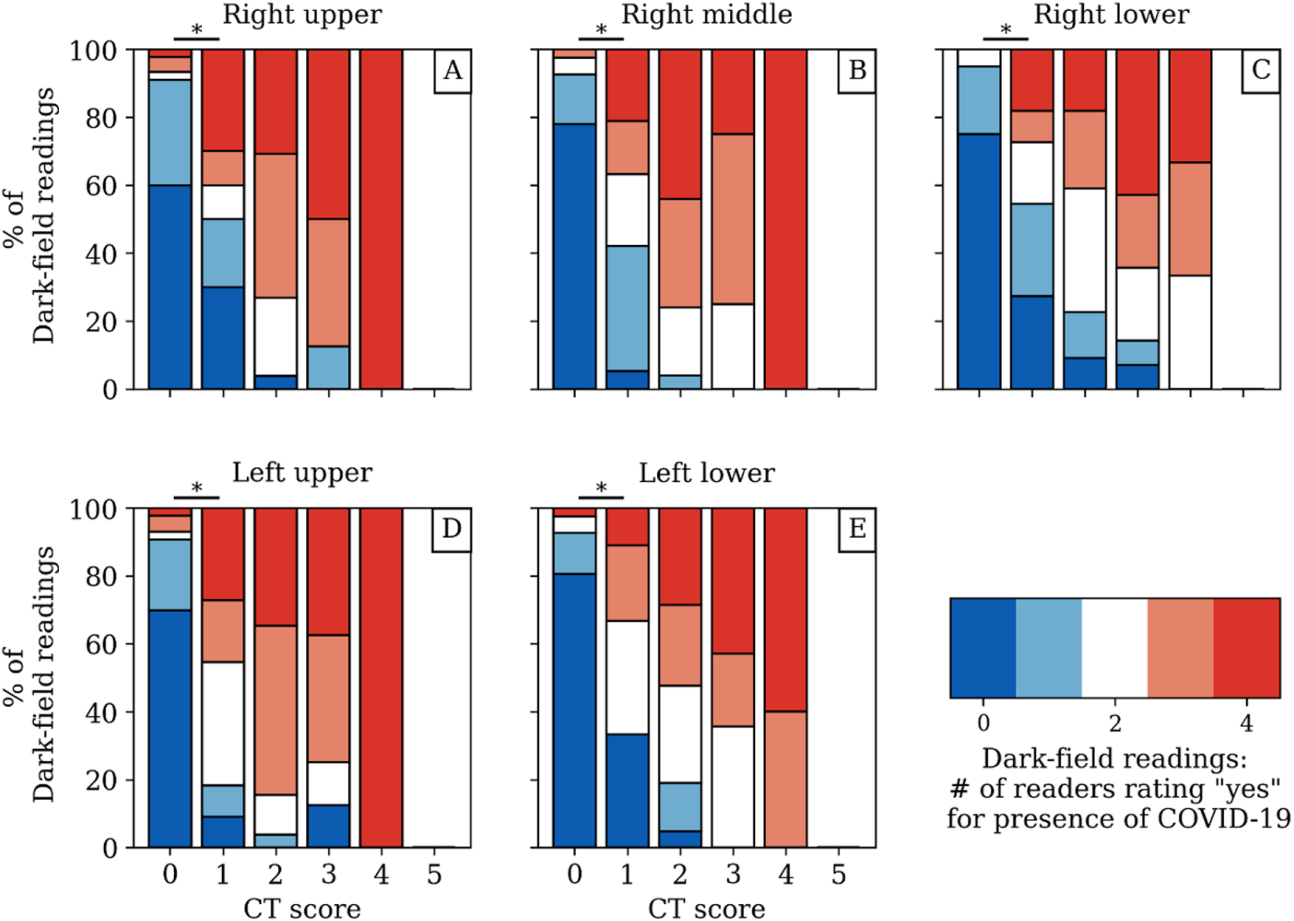
Dark-field-based readings for the presence of COVID-19-associated changes compared to the respective CT-based lobar visual COVID-19 severity grading from CT for each zone/lobe individually **(A–E)** with respect to the agreement between readers. While the red and blue coloring mark a unanimous rating, the other colors correspond to different disagreement scenarios among readers. The asterisk (*) marks a significant difference (*p* < .05) between the dark-field-based ratings of the groups with CT score “0” and “1”. The underlying data set can be found in Supplemental Table 2.

The dark-field coefficient was negatively correlated with both the quantitative CT-based COV-I (*r* = −.34, *p* = .001, 95% CI [−.52, −.12]) (Figure 5A) and the total CT score for visual COVID-19 severity grading (*r* = − .44, *p* < .001, 95% CI [−.61, −.25]). The total visual dark-field score for the presence of COVID-19 was correlated positively with the total CT score for visual COVID-19 severity grading (*r* = .85, *p* < .001, 95% CI [.81, .93]) (Figure 5B). On a lobe level, higher CT-based visual COVID-19 severity grading corresponded to a higher rate of COVID-19 positive readings in the respective zone on dark-field images (Figure 6). In all lobes/zones, compared to the group with CT score “0”, we found significantly more positive rates for dark-field readings already for the group with CT score “1”. An overview of all dark-field and CT readings is provided in Supplementary Table 2.

## 4 Discussion

In this study, we investigated both the qualitative and quantitative aspects of dark-field chest radiographs in individuals with COVID-19 pneumonia, comparing them to CT imaging results. Unlike images from healthy subjects, those of patients with COVID-19 pneumonia exhibited reduced dark-field signal intensity and a patchy, uneven lung appearance. The areas of diminished signal intensity in dark-field images aligned closely with affected regions identified on CT scans. There was a negative correlation between the dark-field coefficient (m^−1^) and both the quantitative CT-based COVID-19 index (*r* = −.34, *p* = .001) and the overall visual severity grading from CT (*r* = −.44, *p* < .001). Conversely, the total visual dark-field score showed a strong positive correlation with the overall visual severity grading of COVID-19 from CT images (*r* = .85, *p* < .001).

Dark-field image characteristics in humans have already been investigated in previous studies. In a cohort of 40 healthy humans, Gassert et al. (19) have described the qualitative characteristics of dark-field chest radiographs. Additionally, the quantitative dark-field coefficient was assessed and compared against demographic factors such as sex, age, weight, and height. Willer et al. (18) explored the use of dark-field chest radiography in individuals with COPD, demonstrating its effectiveness in detecting and grading emphysema through a reader study. In a separate study, the quantitative dark-field coefficient was applied to patients with emphysema to further evaluate its utility: Urban et al. (20) observed that emphysema results in decreased signal intensity on dark-field chest radiographs because of the reduced number of tissue-air interfaces. Additionally, the regions of focal signal intensity loss on dark-field images closely matched the emphysematous areas identified on CT scans. Other pulmonary pathologies dark-field imaging was suggested for are lymphangioleiomyomatosus (28) and combined pulmonary fibrosis and emphysema (29).

In those previously mentioned studies, the change of dark-field signal was caused by a smaller number of alveolar walls traversed by the x-ray beam resulting in fewer interfaces and thus less small-angle scattering. In acute COVID-19 pneumonia, however, the number of alveolar walls remains the same. Instead, alveoli are filled with inflammatory fluid, debris, and cells, which also results in fewer tissue-air-interfaces and a reduced dark-field signal. Thus, a lower dark-field signal can be the result of both the destruction of alveolar walls as in emphysema as well as of the filling of alveoli with inflammatory fluid as in pneumonia.

Consistent with findings in emphysema patients, our study revealed that the dark-field appearance in individuals with COVID-19 pneumonia was markedly inhomogeneous and patchy, in contrast to the uniform dark-field signal intensity observed in healthy lungs. This inhomogeneity was attributed to the presence of focal infiltrates. The strong correlation between the total visual dark-field score and the total visual CT score shows that infiltrates in CT images were also identified in the dark-field images. This finding is supported by the strong correlation between the CT-based COVID index and the total visual dark-field score.

The strong correlation between the total visual CT score and the quantitative CT-based COVID-19 index indicates that the introduced COVID-19 index accurately represents the presence of COVID-19 pneumonia. However, the objective dark-field coefficient only shows weak correlation with other metrics. While the dark-field coefficient is a global metric derived from the entire lung, COVID-associated changes might only affect a (very) small part of the lungs and thus only cause small changes in the dark-field coefficient. This might be the reason for the rather low correlation between dark-field coefficient and other quantities.

While this study investigated dark-field imaging for inflammatory processes in humans, our results are in line with previous animal studies. Hellbach et al. (17) showed that acute lung inflammation in a mouse model leads to a strongly reduced dark-field signal. Also, several studies have shown that dark-field imaging allows for the assessment of radiation-induced lung changes, both in a two-dimensional (30) and a three-dimensional (13, 31) setting.

In the broader context of COVID-19 imaging, our findings complement existing research on CT imaging, which remains the gold standard for visualizing pulmonary involvement due to its high sensitivity for detecting ground-glass opacities and consolidations (4). Furthermore, studies such as by Bai et al. (32) have shown the utility of CT in differentiating COVID-19 pneumonia from other viral pneumonias, highlighting the importance of specific imaging biomarkers. Compared to these modalities, dark-field chest radiography offers a novel approach by directly assessing structural alveolar changes through small-angle x-ray scattering. This technique could serve as a lower-radiation alternative, particularly in settings where CT is not readily available or in populations requiring dose minimization, such as pediatric or serial imaging scenarios. Recent AI-based methods, as described by Li et al. (33), have also shown promise in automating COVID-19 diagnosis using chest x-rays, which, when combined with dark-field imaging, could further enhance diagnostic accuracy and efficiency. Our study bridges these advancements by demonstrating the correlation of dark-field findings with established CT metrics, providing a pathway for integrating novel imaging approaches with existing clinical workflows. A state-of-the-art (SOTA) table comparing methodologies, accuracy, assumptions, and limitations across different imaging modalities can be found under Table 2.

**Table 2:** SOTA table.

Study	Modality	Accuracy	Limitations	Assumptions
Wong et al. (4)	CT	High sensitivity	High radiation dose	Ground-glass opacities indicate COVID-19
Bai et al. (32)	CT	Differentiates pneumonia types	Cost-intensive, limited access	Viral pneumonia imaging is standardized
Li et al. (33)	AI-enhanced radiography	Moderate accuracy	Requires extensive datasets	AI models generalize well across populations
Rubin et al. (34)	Conventional radiography	Moderate sensitivity	Lower resolution than CT	Visual patterns indicate disease severity
Present study	Dark-field radiography	High correlation	Excludes severely ill patients	Patchy patterns indicate COVID-19 pneumonia

This study has limitations. For eight COVID-19 patients, the lateral image could not be acquired due to the patient's condition and therefore determination of the lung volume was not possible. Those patients were only included in the reader study but not in the quantitative analysis. While a negative COVID-19 PCR test was an exclusion criterion, a positive test was not necessarily needed for study inclusion. Also, except for pneumothorax, pleural effusion, and lung cancer, exclusion criteria did not comprise other pulmonary diseases that might potentially influence the dark-field signal. Furthermore, we did not differentiate between ground-glass opacities and consolidations, nor did we compare COVID-19 pneumonia to pneumonia other than COVID-19. This needs to be the subject of future studies. Moreover, the requirement for patients to stand upright and hold their breath likely led to the inclusion of less severely ill patients in the study.

In conclusion, we found that COVID-19 pneumonia leads to a reduced signal intensity on dark-field chest radiographs and that focal signal losses in dark-field radiography correspond well to focal COVID-19-associated lung changes in CT imaging. This suggests the capability of the method for the assessment of COVID-19 pneumonia and underlines its potential for the diagnosis of other lung diseases that impair the alveolar integrity.

## Data Availability

The raw data supporting the conclusions of this article will be made available by the authors, without undue reservation.
