# Supplementary material for: Comparison of dark-field chest radiography and CT for the assessment of COVID-19 pneumonia
Source: Front Radiol. 2025 Jan 14;4:1487895. doi: 10.3389/fradi.2024.1487895 (PMC11772474; doi:10.3389/fradi.2024.1487895)
Supplement: Supplementary file 1 [file Datasheet1.pdf]

Supplementary Material

1 Supplementary Tables

Supplemental Table 1: Inter-rater reliability expressed with Fleiss' Kappa

CT images

	Left top	Left bottom	Right top	Right middle	Right bottom
κ	0.58	0.57	0.50	0.48	0.60

Dark-field images

	Left upper	Left lower	Right upper	Right middle	Right lower
κ	0.55	0.55	0.51	0.51	0.42

Supplemental Table 2: Dark-field reading results versus CT reading results on a zone-/lobe-level

Right upper

# of readers rating "yes" for presence of COVID-19	CT score						*Sum*
	0	1	2	3	4	5	
0	27	4	1	0	0	0	33
1	14	2	0	1	0	0	17
2	1	2	6	0	0	0	9
3	2	2	12	3	0	0	19
4	1	5	9	5	2	0	22
Sum	45	15	28	9	1	0	98

Right middle

# of readers rating "yes" for presence of COVID-19	CT score						
	0	1	2	3	4	5	*Sum*
0	32	3	0	0	0	0	35
1	6	8	2	0	0	0	17
2	2	4	5	1	0	0	12
3	1	4	8	2	0	0	15
4	0	5	12	2	1	0	21
Sum	41	24	27	5	1	0	98

Right lower

# of readers rating "yes" for presence of COVID-19	CT score						*Sum*
	0	1	2	3	4	5	
0	30	3	3	1	0	0	38
1	8	3	3	1	0	0	15
2	2	3	11	3	1	0	20
3	0	1	6	3	1	1	12
4	0	2	5	6	1	0	15
Sum	40	12	28	14	4	1	98

Left upper

# of readers rating "yes" for presence of COVID-19	CT score						*Sum*
	0	1	2	3	4	5	
0	30	1	1	1	0	0	34
1	9	1	1	0	0	0	11
2	1	5	3	1	0	0	10
3	2	2	13	3	0	0	21
4	1	5	12	4	2	0	24
Sum	43	14	30	9	2	0	98

Left lower

# of readers rating "yes" for presence of COVID-19	CT score						*Sum*
	0	1	2	3	4	5	
0	33	3	3	0	0	0	41
1	5	0	3	0	0	0	8
2	2	3	6	5	0	0	16
3	0	2	6	4	2	0	14
4	1	1	8	6	4	1	21
Sum	41	10	26	16	6	1	98
